# Improving outcomes of liver resection for hepatocellular carcinoma associated with portal vein tumor thrombosis over the evolving eras of treatment

**DOI:** 10.1186/s12957-021-02425-w

**Published:** 2021-10-26

**Authors:** Yu-Chao Wang, Jin-Chiao Lee, Tsung-Han Wu, Chih-Hsien Cheng, Chen-Fang Lee, Ting-Jung Wu, Hong-Shiue Chou, Kun-Ming Chan, Wei-Chen Lee

**Affiliations:** Department of General Surgery and Chang Gung Transplantation Institute, Chang Gung Memorial Hospital at Linkou, Chang Gung University College of Medicine, Taoyuan, Taiwan

**Keywords:** Hepatocellular carcinoma, Liver resection, Portal vein tumor thrombosis, Outcomes, Era

## Abstract

**Background:**

The outcomes and management of hepatocellular carcinoma (HCC) have undergone several evolutionary changes. This study aimed to analyze the outcomes of patients who had undergone liver resection for HCC with portal vein tumor thrombosis (PVTT) in terms of the evolving era of treatment.

**Materials and methods:**

A retrospective analysis of 157 patients who had undergone liver resection for HCC associated with PVTT was performed. The outcomes and prognostic factors related to different eras were further examined.

**Results:**

Overall, 129 (82.1%) patients encountered HCC recurrence after liver resection, and the median time of recurrence was 4.1 months. Maximum tumor size ≥ 5 cm and PVTT in the main portal trunk were identified as the major prognostic factors influencing HCC recurrence after liver resection. Although the recurrence-free survival had no statistical difference between the two eras, the overall survival of patients in the second era was significantly better than that of the patients in the first era (*p* = 0.004). The 1-, 2-, and 3-year overall survival rates of patients in the second era were 60.0%, 45.7%, and 35.8%, respectively, with a median survival time of 19.6 months.

**Conclusion:**

The outcomes of HCC associated with PVTT remain unsatisfactory because of a high incidence of tumor recurrence even after curative resection. Although the management and outcomes of patients with HCC and PVTT have greatly improved over the years, surgical resection remains an option to achieve a potential cure of HCC in well-selected patients.

## Introduction

Hepatocellular carcinoma (HCC) is the most common primary liver cancer and the third leading cause for cancer-related deaths worldwide [1, 2]. Specifically, the majority of patients with HCC are detected at an advanced stage due to a lack of specific symptoms in the clinical scenario. Additionally, advanced HCC is usually characterized by the early formation of portal vein tumor thrombosis (PVTT) that approximately occurs in 35 to 50% of the patients [3, 4]. PVTT carries a high risk of extensive intrahepatic metastasis and rapid deterioration of liver function related to portal inflow compromise, and thus remains one of the most negative prognostic factors leading to a dismal outcome [5–7].

Generally, the majority of HCC patients with PVTT have no optimal treatment options, in which the overall survival is approximately 2 to 4 months for patients without any treatment [5, 8]. Although surgical resection remains the mainstay curative treatment for HCC, the patients who are eligible for liver resection for HCC associated with PVTT are few. Nowadays, along with the advancement of surgical instruments and perioperative patient care, liver resection is considered as a safety procedure even in HCC patients with PVTT [9, 10]. However, the oncologic outcome and patient survival are both of the utmost importance regardless of the therapeutic approach given for patients with HCC. Therefore, this study gathered data on patients who had advanced HCC associated with PVTT to evaluate the therapeutic outcome and prognosis of liver resection. Additionally, the treatment of advanced HCC has significantly changed because of the introduction of a novel therapeutic regimen, such as multikinase inhibitors, over the last decade. Therefore, the patient cohort was also grouped and compared in different eras based on the introduction of sorafenib to evaluate the evolution of the outcomes over the years.

## Materials and method

### Patients

The patients who had received curative liver resection for primary HCC associated with PVTT at the Department of General Surgery, Chang Gung Memorial Hospital at Linkou Medical Center, Taiwan, during the period from January 2003 to December 2017 were retrospectively reviewed. Under the approval of the Institutional Review Board, a total of 203 patients were thoroughly reviewed. All the patients underwent pathological confirmation of HCC. The extent of the PVTT was determined by a preoperative imaging examination and/or the pathological assessment of the resected liver specimen. The degree of PVTT was classified according to the classification system proposed by the Liver Cancer Study Group of Japan [11]. Briefly, Vp1 was PVTT confined to the distal portal branches; Vp2 represented PVTT that extended to second-order portal branches; Vp3 was PVTT involved in the first-order portal branches; and Vp4 was PVTT detected in the main portal trunk.

A standard data collection through the reviewing of medical records was completed for all the patients in this study. Comprehensive information including the demographic characteristics, preoperative laboratory tests, operative factors, histologic features, cancer-related postoperative follow-up, and status of survival were collected for analysis. In order to analyze the impact of HCC with dominant PVTT, the patients with Vp1 invasion (*n* = 46) were excluded from this study. The remaining 157 patients were included in the study for analysis. Additionally, the patient cohort was arbitrarily divided into 2 subgroups based on the introduction of sorafenib in the institute as era 1 (2003–2008, pre-2009) and era 2 (2009–2017, post-2009) for comparison.

### Preoperative evaluation

The evaluation and diagnosis of HCC were generally correlated with the guidelines proposed by the European Association for the Study of the Liver (EASL) and the American Association for the Study of Liver Diseases (AASLD) [12, 13]. Generally, the imaging examination of either dynamic liver computed tomography (CT) or magnetic resonance imaging (MRI) and the measurement of serum α-fetoprotein (AFP) are mandatory. Additionally, the complete preoperative evaluation, including medical history, physical examination, and baseline laboratory tests, was examined as a cancer-specific evaluation. The assessment of the eligibility for liver resection was determined mainly based on the balance of the patient’s performance, liver functional reserve, and tumor features. Additionally, the indocyanine green (ICG) retention rate at 15 min was also assessed to determine the extent of the liver resection as proposed by the Makuuchi algorithm [14]. The extent of the liver resection was defined as the Couinaud’s classification of liver segments.

### Postoperative follow-up

After the operation, all the patients were regularly followed up for the surveillance of HCC recurrence and survival until death or the end of the present study. During the follow-up period, the clinical assessments, including serum biochemical laboratory tests, AFP measurement, and abdominal ultrasonography were monitored at monthly intervals in the initial 3 months and every 3 months thereafter. CT and/or MRI were performed on an annual basis or whenever suspicious of HCC recurrence.

### Outcomes and statistical analysis

The outcomes were measured in terms of HCC recurrence-free survival (RFS) and the patient’s overall survival (OS). RFS was defined as the period between the dates of liver resection to the date of the detection of HCC recurrence. OS was estimated from the date of liver resection until the date of death or last follow-up. The categorical variables were assessed using the chi-square or Fisher’s exact test as appropriate, and the independent *t* test was used for continuous data. AFP was set at 400 ng/ml as a cutoff value for risk stratification as previous studies [15, 16], because receiver operating characteristic (ROC) analysis was not able to identify an optimal cut-off value in this study. Univariate analyses of the variables were conducted using the Cox regression proportional hazards model to identify the potential prognostic factors of RFS and OS. Subsequently, all significant prognostic factors in the univariate analysis were selected for multivariate analysis. All statistical analyses were performed using 20.0 edition SPSS statistical software (SPSS, Inc., Chicago, IL, USA) for Windows. A *p* value of less than 0.05 was considered to be statistically significant.

## Results

### Patient characteristics

Of the 157 patients, there were 137 (87.3%) men and 20 (12.7%) women with ages ranging from 28.5 to 83.0 years at the time of liver resection. Based on the classification of PVTT, tumor thrombus was detected in Vp2, Vp3, and Vp4 for 63 (40.1%), 80 (51.0%), and 14 (8.9%) patients, respectively. Overall, 15 (9.6%) patients were alive without evidence of HCC, 15 (9.6%) patients were still alive but with HCC, and 127 (80.8%) patients died at the end of this study. The patient cohort was divided into 2 subgroups according to the arbitrary time point (January 1, 2009): the first period was defined as pre-2009 (era 1, *n* = 52), and the later period was defined as post-2009 (era 2, *n* = 105). Table 1 summarizes and compares the clinicopathological features of the patients of era 1 and era 2. There were significant differences in the hepatitis virus distribution, treatment modality of the first postoperative HCC recurrence, additional tyrosine kinase inhibitors (TKIs) for recurrent HCC, and the current status of patient outcomes between the 2 groups. The patients of era 1 had a higher ratio of hepatitis B virus carriers, and a significantly higher percentage of patients were given TKI after HCC recurrence in era 2. Specifically, 12 patients who accounted for 11.4% in era 2 were cured of HCC by the end of this study (*p* = 0.009). However, there was no significant difference related to the type of PVTT and extent of liver resection between the 2 groups. Although the surgical mortality seems to be higher in era 2, no significant difference was observed as compared with that in era 1.

**Table 1:** Clinical characteristics of patients undergoing liver resection for hepatocellular carcinoma with major portal vein tumor thrombosis

Characteristics	Era 1 *n* = 52	Era 2 *n* = 105	*p* value
Age (years), median (range)	54.5 (29.9–76.4)	58.0 (28.5–83.0)	0.253
Gender			
Male	48 (92.3%)	89 (84.8%)	0.182
Female	4 (7.7%)	16 (15.2%)	
Hepatitis status			
HBV(+)	40 (76.9%)	57 (54.3%)	0.013
HCV(+)	7 (13.5%)	20 (19%)	
HBV and HCV(+)	2 (3.8%)	2 (1.9%)	
None	3 (5.8)	26 (24.8)	
Maximum tumor size (cm)			
Median, range	8.0 (1.5–25.0)	8.0 (1.6–19.0)	0.482
Portal vein tumor thrombosis			
Vp2	23(44.2%)	40 (38.1%)	0.446
Vp3	23 (44.2%)	57 (54.3%)	
Vp4	6 (11.5%)	8 (7.6%)	
Preoperative treatment			
TACE/TKIs	0	1 (1.0%)	0.096
TACE	0	5 (4.8%)	
TKIs	0	1 (1.0%)	
No	52 (100%)	98 (93.2%)	
Extent of liver resection			
< 3 segments	9 (17.3%)	13 (12.4%)	0.403
≥ 3 segments	43 (82.7%)	92 (87.6%)	
HCC recurrence			
Intrahepatic only	7 (13.5%)	15 (14.3%)	0.821
Intrahepatic and systemic	30 (57.7%)	51 (48.6%)	
Systemic	6 (11.5%)	20 (19.0%)	
No	9 (17.3%)	19 (18.1%)	
Treatment of recurrent HCC*			
Surgical resection	1 (2.3%)	5 (5.8%)	0.01
Locoregional therapy	27 (62.8%)	57 (66.3%)	
Systemic chemotherapy	2 (4.7%)	8 (9.3%)	
Others	1 (2.3%)	7 (8.1%)	
No	12 (27.9%)	9 (10.5%)	
Additional TKIs for recurrence*			
Yes	4 (8.9%)	39 (45.3%)	0.0001
No	39 (91.1%)	47 (54.7%)	
Hospital mortality	2 (3.8%)	7 (6.7%)	0.719
Outcomes			
Died of HCC	43 (82.7%)	69 (63.8%)	0.009
Death unrelated to HCC	4 (7.7%)	2 (1.9%)	
Alive with HCC	0	15 (14.3%)	
Alive without HCC	3 (5.8%)	12 (11.4%)	

*HBV*, hepatitis B virus; *HCV*, hepatitis C virus; *Vp2*, portal vein tumor thrombosis in second-order portal branches; *Vp3*, portal vein tumor thrombosis in first-order portal branches; *Vp4*, portal vein tumor thrombosis in the main portal trunk; *TACE*, transcatheter arterial chemoembolization; *HCC*, hepatocellular carcinoma; *TKI*, tyrosine kinase inhibitor; asterisk (*) represents percentage within recurrence

### Prognostic factors affecting outcomes

Overall, 129 (82.1%) patients had HCC recurrence after liver resection, in which 22 patients (14.0%) had intrahepatic recurrence only and 107 patients (68.1%) showed an additional systemic spread as shown in Table 1. The clinicopathological factors affecting HCC recurrence were analyzed for patients after liver resection. Table 2 shows the univariate and multivariate analysis of the prognostic factors for all the patients. Univariate analysis showed that hepatitis B virus (HBV) positivity, resection margin < 0.5 cm, maximum tumor size ≥ 5 cm, and PVTT were significant factors. Subsequently, multivariate regression analysis of these factors identified 3 risk factors: resection margin < 0.5 cm (hazard ratio (*HR*) = 1.66, *p* = 0.023), maximum tumor size ≥ 5 cm (*HR* = 1.61, *p* = 0.044), and Vp4 tumor thrombosis (*HR* = 2.21, *p* = 0.018), as affecting the HCC recurrence for all patients after liver resection.

**Table 2:** Univariate and multivariate analyses of clinicopathological factors affecting HCC recurrence of patients after liver resection

	Univariate			Multivariate	
	Median RFS months	95% CI	*p* value	Hazard ratio (95% CI)	*p* value
Age (years)			0.832	-	
< 60	3.91	2.92–4.90			
≥ 60	4.31	2.15–6.46			
Gender			0.554	-	
Female	3.52	1.93–5.10			
Male	4.08	2.94–5.22			
Hepatitis B virus			0.023		
Positive	3.09	2.62-3.56		1.32 (0.91–1.93)	0.144
Negative	5.82	1.69–9.95		1	
Hepatitis C virus			0.149	-	
Positive	5.72	0.27–11.17			
Negative	3.42	2.66–4.18			
ICG 15 min (%)			0.936	-	
< 10	3.35	1.94-4.76			
≥ 10	3.45	3.26–3.64			
AFP (ng/ml)			0.426	-	
≥ 400	5.82	4.12–7.52			
< 400	3.03	2.35–3.70			
Tumor capsule			0.050		
Presence	5.20	4.11–6.28		1	
Absence	2.89	1.98–3.80		1.27 (0.87–1.87)	0.218
Satellite nodule			0.888	-	
Presence	3.55	2.42–4.68			
Absence	4.57	2.57–6.57			
Extent of liver resection			0.662	-	
< 3 segments	3.03	2.00–4.05			
≥ 3 segments	4.08	2.92–5.23			
Resection margin			0.024		
< 0.5 cm	2.07	0.35–3.79		1.66 (1.07–2.56)	0.023
≥ 0.5 cm	4.57	3.37–5.77		1	
Maximum tumor size			0.009		
< 5cm	11.47	3.01–19.94		1	
≥ 5cm	3.39	2.65–4.12		1.61 (1.01–2.60)	0.044
Portal vein invasion			0.006		
Vp2	4.87	2.44–7.29		1	
Vp3	4.18	2.83–5.52		1.16 (0.79–1.69)	0.448
Vp4	1.61	0.61–2.62		2.21 (1.15–4.24)	0.018

*HCC*, hepatocellular carcinoma; *RFS*, recurrence-free survival; *CI*, confidence interval; *ICG*, indocyanine green; *Vp2*, portal vein tumor thrombosis in second-order portal branches; *Vp3*, portal vein tumor thrombosis in first-order portal branches; *Vp4*, portal vein tumor thrombosis in the main portal trunk

An analysis of the risk factors for HCC recurrence according to the two different eras is summarized in Table 3. Univariate analysis showed that the width of the resection margin and PVTT were the significant factors in era 1. However, none of the two factors were independent risk factors after subsequent multivariate regression analysis. In era 2, two factors, including maximum tumor size ≥ 5 cm and PVTT, were identified by univariate analysis. Multivariate regression analysis further confirmed that both factors, maximum tumor size ≥ 5 cm (*HR* = 1.92, *p* = 0.019), and Vp4 tumor thrombosis (*HR* = 2.98, *p* = 0.041) significantly influenced the recurrence of HCC in the era 2 group.

**Table 3:** Univariate and multivariate analyses of clinicopathological factors affecting HCC recurrence of patients after liver resection in different eras

Factors	Era 1					Era 2				
	Univariate			Multivariate		Univariate			Multivariate	
	Median RFS months	95% CI	*p* value	Hazard ratio (95% CI)	*p* value	Median RFS months	95% CI	*p* value	Hazard ratio (95% CI)	*p* value
Age (years)			0.868	-				0.847	-	
< 60	3.52	2.30–4.74				3.91	2.34–5.48			
≥ 60	3.45	2.03–4.88				5.26	2.13–8.39			
Gender			0.976	-				0.539	-	
Female	3.50	0.01–9.83				3.29	1.23–5.35			
Male	3.46	2.41–4.49				4.57	3.03–6.11			
Hepatitis B virus			0.524	-				0.071	-	
Positive	3.35	2.75–3.96				2.96	1.97–3.94			
Negative	5.29	2.98–7.60				6.18	1.87–10.49			
Hepatitis C virus			0.293	-				0.444	-	
Positive	9.96	2.96–16.96				5.72	4.04–7.40			
Negative	3.35	2.82–3.89				3.55	1.92–5.18		-	
ICG 15 min (%)			0.720	-				0.984		
< 10	4.31	2.60–6.02				3.35	0.84–5.87			
≥ 10	3.52	2.68–4.36				3.42	2.14–4.70			
AFP (ng/ml)			0.931	-				0.328	-	
≥ 400	3.39	2.78–4.00				2.56	1.81–3.32			
< 400	4.73	2.68–6.78				6.18	4.02–8.34			
Tumor capsule			0.245	-				0.153	-	
Presence	4.37	2.72–7.87				5.20	3.83–6.56			
Absence	3.45	2.77–4.14				2.14	1.34–2.93			
Satellite nodule			0.511	-				0.663	-	
Presence	3.52	2.39–4.65				4.18	1.56–6.79			
Absence	3.45	0.65–6.25				4.57	2.18–6.96			
Extent of liver resection			0.593	-				0.825	-	
< 3 segments	2.89	1.21–4.58				4.87	0.85–8.88			
≥ 3 segments	4.08	2.87–5.29				4.01	2.34–5.68			
Resection margin			0.012		0.062			0.304	-	
< 0.5 cm	1.38	0.43–2.33		2.01 (0.97–4.18)		2.99	0.46–5.52			
≥ 0.5 cm	4.31	2.68–5.94		1		4.87	3.21–6.52			
Maximum tumor size			0.717	-				0.006		
< 5cm	3.45	0.01–9.61				12.82	0.01–27.85		1	
≥ 5cm	3.52	2.39–4.65				3.35	2.49–4.22		1.92 (1.11–3.31)	0.019
Portal vein invasion			0.044					0.036		
Vp2	3.35	2.64–4.07		1		5.82	3.93–7.71		1	
Vp3	4.73	2.19–7.28		0.95 (0.49–1.81)	0.864	3.91	2.44–5.39		0.88 (0.47–1.67)	0.705
Vp4	2.70	0.01–5.80		2.21 (0.75–6.48)	0.150	1.61	0.01–3.21		2.98 (1.04–8.48)	0.041

*HCC*, hepatocellular carcinoma; *RFS*, recurrence-free survival; *CI*, confidence interval; *ICG*, indocyanine green; *Vp2*, portal vein tumor thrombosis in second-order portal branches; *Vp3*, portal vein tumor thrombosis in first-order portal branches; *Vp4*, portal vein tumor thrombosis in the main portal trunk

### Survival analysis

**Fig. 1:**
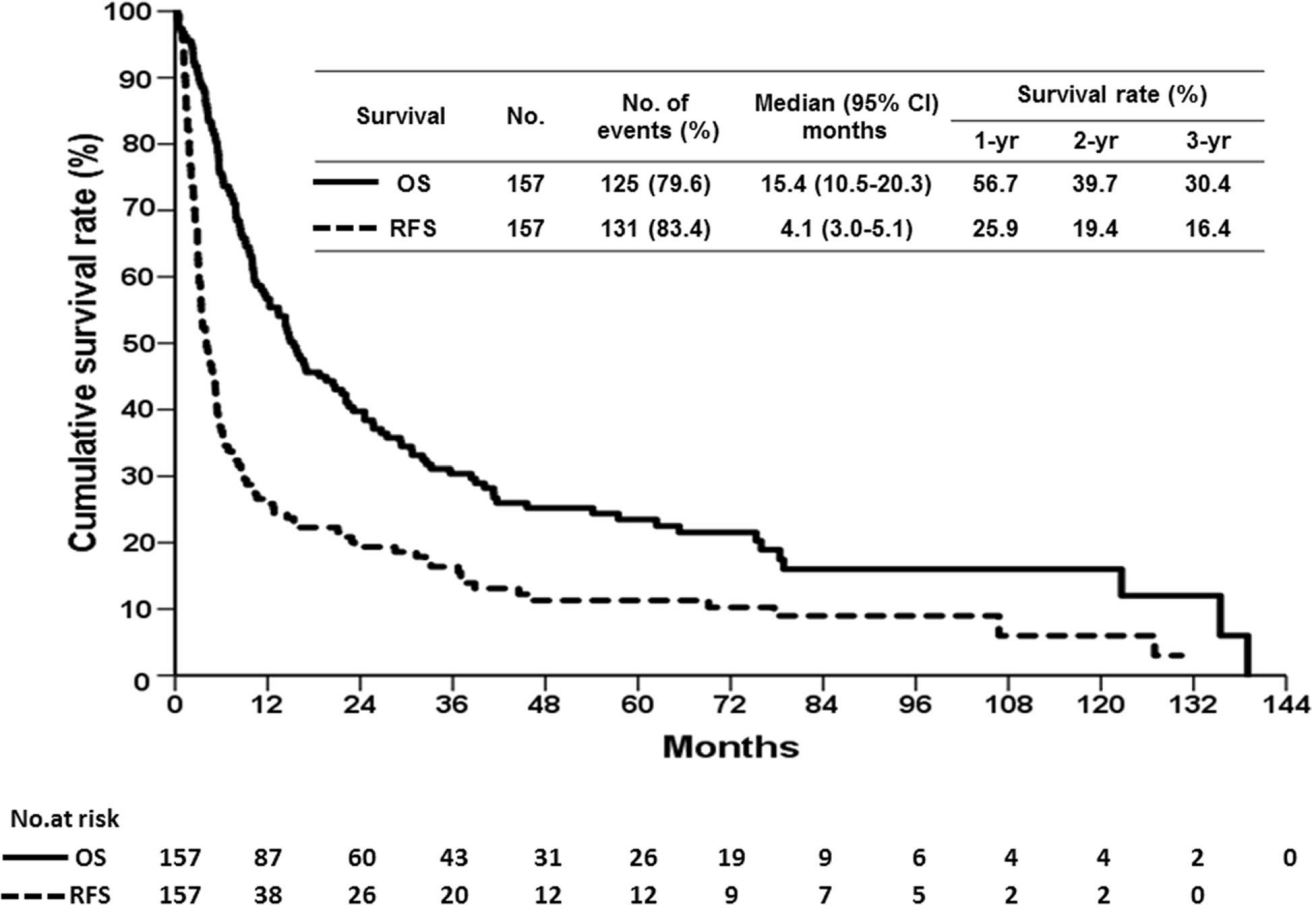
Long-term cumulative recurrence-free survival (RFS) and overall survival (OS) curves of patients undergoing liver resection for hepatocellular carcinoma associated with portal vein tumor thrombosis

Overall, the median follow-up period for the included patients was 14.8 months (range, 0.2 to 139.0 months) after liver resection. The survival curves of patients in this study are illustrated in Fig. 1. The median time of the HCC recurrence after liver resection was 4.1 months, and the RFS for 1, 2, and 3 years was 25.9%, 19.4%, and 16.4%, respectively. The median time of the patient survival was 16.4 months, and the OS for 1, 2, and 3 years was 56.7%, 39.7%, and 30.4%, respectively. Furthermore, the outcomes of the patients were compared according to the two different eras.

**Fig. 2:**
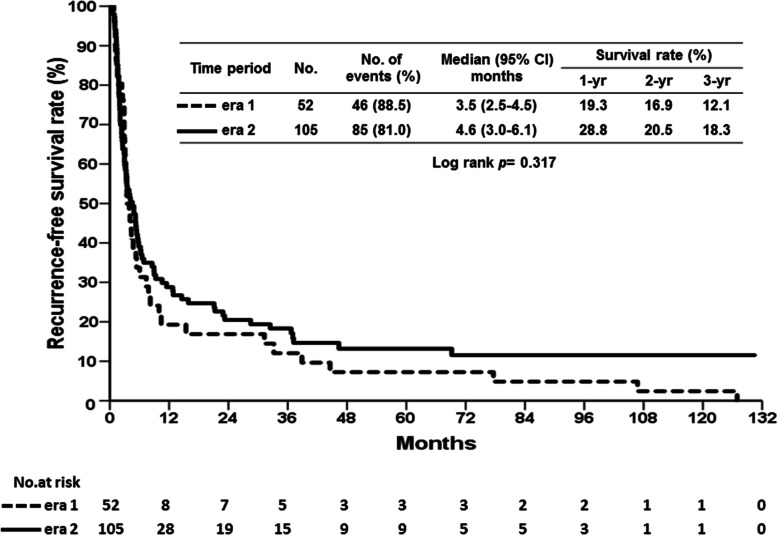
Cumulative recurrence-free survival (RFS) curves of patients who underwent liver resection for hepatocellular carcinoma associated with portal vein tumor thrombosis according to the two eras. The comparison of RFS curves between the two eras had no significant difference (*p* = 0.317)

**Fig. 3:**
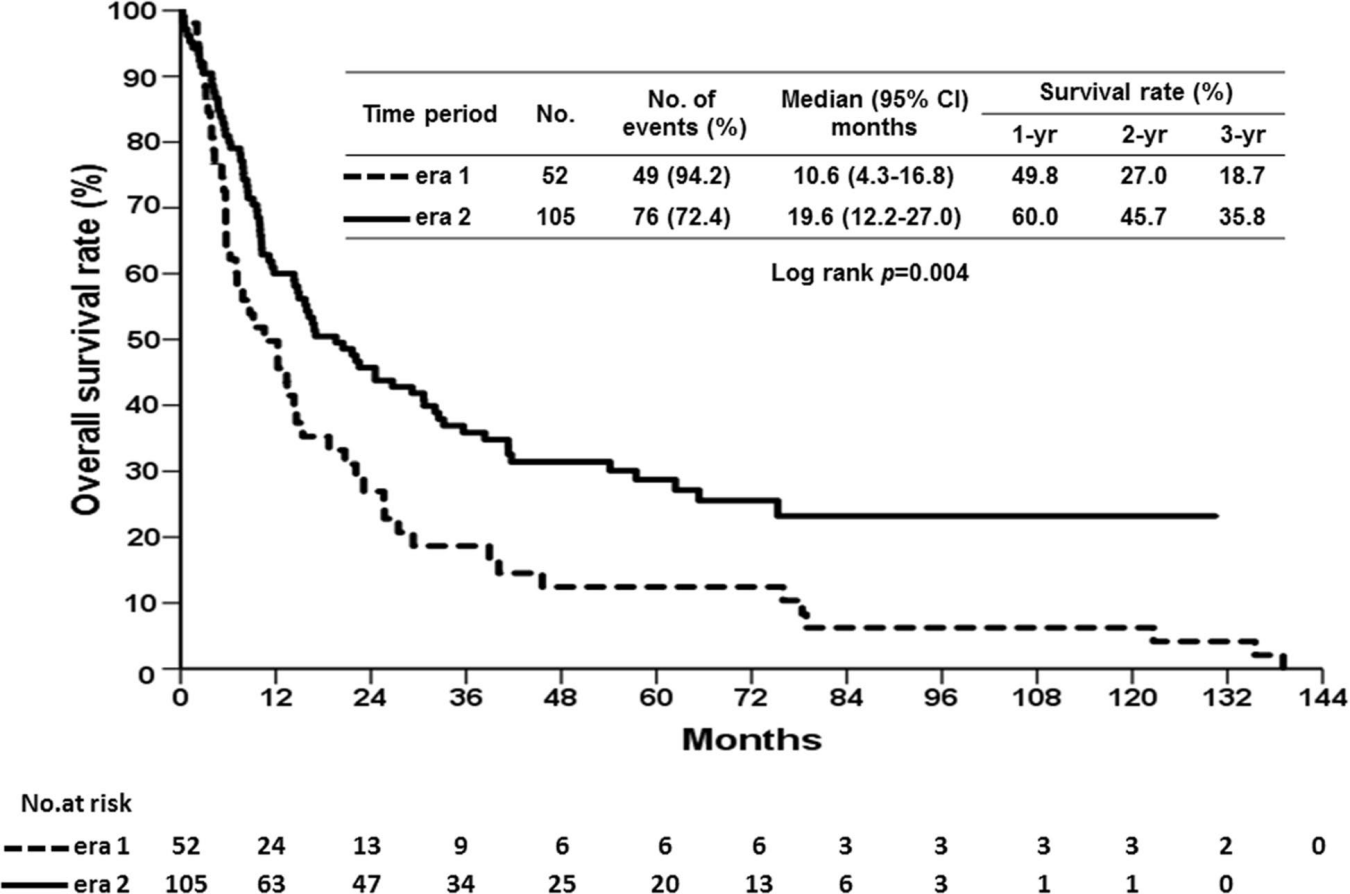
Cumulative overall survival (OS) curves of patients who underwent liver resection for hepatocellular carcinoma associated with portal vein tumor thrombosis according to the two eras. The OS rate in era 2 was better than the OS rate in era 1 (*p* = 0.004)

The median duration of follow-up for patients in era 1 and era 2 after liver resection were 8.9 months (range, 0.2 to 139.0 months) and 19.6 months (range, 0.3 to 130.4 months), respectively. Although the RFS curve of patients in era 2 was better than that of patients in era 1, no statistical difference was observed between the two eras (Fig. 2, *p* = 0.317). The median time of HCC recurrence was 3.5 months in era 1, and the 1-, 2-, and 3-year RFS rates were 19.3%, 16.9%, and 12.1%, respectively. The median time of HCC recurrence in era 2 was 4.6 months, and the 1-, 2-, and 3-year RFS rates were 28.8%, 20.5%, and 18.3%, respectively. However, the OS was significantly different between the two eras. The OS of patients in era 2 was significantly better than that of patients in era 1 (Fig. 3, *p* = 0.004). The era 2 patients had a better OS curve, and the 1-, 2-, and 3-year survival rates were 60.0%, 45.7%, and 35.8%, respectively, with a median survival time of 19.6 months. The 1-, 2-, and 3-year OS rates in era 1 patients were 49.8%, 27.0%, and 18.7%, respectively, with a median survival time of 10.6 months.

## Discussion

Portal vein invasion has been recognized as a negative prognostic factor in hepatocellular carcinoma treatment [17–19]. However, due to inconsistent surgical outcomes and various clinicopathological factors, the optimization of a surgical plan for advanced HCC with PVTT is still controversial. HCC associated with PVTT causes a more rapid intrahepatic metastasis and liver dysfunction leading to dismal outcomes as compared with those without portal vein invasion [20]. Nonetheless, surgical resection remained as one of the curative therapeutic options in certain patients [21–24]. The study thus collected our experience and analyzed the differences during the evolving timeframes. Despite the high incidence of postoperative HCC recurrences, the overall survival of these patients showed much progress during recent years.

Generally, HCC with major vascular invasion had been proposed as a systemic disease and is not recommended for surgical resection in most treatment guidelines. Specifically, HCC with portal vascular invasion is considered as an advanced stage and unresectable HCC according to the Barcelona Clinic Liver Cancer (BCLC) staging system. However, the optimal treatment for these patients remains largely controversial. Although the current novel systemic treatment is promising, the outcomes of patients with advanced-stage HCC are still not satisfactory. As such, liver resection may be a feasible approach with a potential cure for patients with HCC and PVTT.

The advancement of anesthesia and surgical techniques and better perioperative patient care has dramatically contributed to the safety of liver resections for HCC during the last few years. As a result, major liver resection has been increasingly performed and is accepted as a safe procedure. Additionally, liver resection with curative intent remains the gold standard for HCC treatment offering the most favorable outcome. Therefore, an aggressive surgical approach could also be considered in selected patients under the circumstance of lacking better therapeutic options for these patients nowadays.

Several important prognostic factors have been well characterized as capable of predicting the outcome of HCC patients after liver resection [18, 25, 26]. However, the study only analyzed a certain population of patients undergoing liver resection, and the prognostic factors identified here might be different from those in previous reports. Generally, the prognostic factors affecting tumor recurrence were mostly related to tumor factors in this study. PVTT might be considered as a major vascular invasion or macrovascular invasion, which is an important factor affecting the outcome of patients undergoing liver resection for primary HCC. Similarly, the extent of PVTT accounted for a significant prognostic factor of HCC recurrence after liver resection for patients with HCC and PVTT. Additionally, the study found that there was no significant difference in the baseline demographic features and clinical conditions between the two eras, revealing no significant alteration in the patient selection for surgical planning during the evolving era. Moreover, a similar RFS between these two eras indicated that the nature of tumor aggressiveness had a much stronger impact on HCC recurrence than any other surgical selection factors.

During the last decade, multi-kinase inhibitors, including sorafenib or lenvatinib, were introduced as the first-line therapeutic options for HCC with macrovascular invasion [20, 27, 28]. Nevertheless, the efficacy of TKI as adjuvant therapy for the postoperative recurrence of advanced HCC is still inconclusive. In the circumstances of a similar recurrence-free survival, the overall survival was shown to be comparatively improved in era 2. This result could possibly indicate that the optimal patient surveillance and treatment of recurrent HCC in the current era exerted a positive impact on the outcome of patients with HCC and PVTT. The implementation of TKI is supposed to play an important role to prolong the overall survival of the cohort in the later era as shown in a previous nationwide population study [29].

However, the study might be limited by its retrospective nature and the small number of patients during a long evolving timeframe. Although generalizations about the small number of patients could not be made easily, several remarkable observations might be helpful in the clinical decision-making of managing patients with HCC and PVTT. Meanwhile, further prospective research on patient selection in terms of the feasibility of liver resection and surgical techniques as well as the outcomes based on HCC with PVTT might be required in order to solve the dilemma.

Importantly, the recent advances of novel therapeutic strategies, such as immunotherapy and/or monoclonal antibody regiments appeared to offer optimistic overall and progression-free survival for patients with advanced HCC [30–32]. Generally, the choice of therapeutic strategy should be individualized on the basis of balance against tumor status, feasibility of liver resection, and host condition for patients with HCC. Therefore, the involvement of a multidisciplinary tumor board is mandatory in order to provide the best therapeutic choice for such complex patients as HCC with PVTT. Additionally, the optimal integration of chemotherapy with liver resection, including the use of preoperative neoadjuvant chemotherapy or postoperative adjuvant chemotherapy, remains to be determined for the patients with advanced HCC and PVTT.

## Conclusion

The outcomes related to HCC associated with PVTT remain unsatisfactory because of a high incidence of tumor recurrence even after curative liver resection. The extent of PVTT is also a poor prognostic factor in these patients. However, surgical resection remains an option to achieve an optimal survival with potential cure of disease in the circumstance of a well-selected patient population due to the lack of better therapeutic options for patients with HCC and PVTT.

## Data Availability

All data generated or analyzed during this study are included in this published article.

## Abbreviations

HCC: Hepatocellular carcinoma
PVTT: Portal vein tumor thrombosis
EASL: European Association for the Study of the Liver
AASLD: American Association for the Study of Liver Diseases
CT: Computed tomography
MRI: Magnetic resonance imaging
AFP: α-Fetoprotein
ICG: Indocyanine green
RFS: Recurrence-free survival
OS: Overall survival
TKI: Tyrosine kinase inhibitor
HBV: Hepatitis B virus
HCV: Hepatitis C virus
BCLC: Barcelona Clinic Liver Cancer
